# Evaluation of a capacity building intervention on malaria treatment for under-fives in rural health facilities in Niger State, Nigeria

**DOI:** 10.1186/s12936-020-03167-y

**Published:** 2020-02-24

**Authors:** Ayodele Jegede, Barbara Willey, Prudence Hamade, Fredrick Oshiname, Daniel Chandramohan, IkeOluwa Ajayi, Catherine Falade, Ebenezer Baba, Jayne Webster

**Affiliations:** 1 Department of Sociology, University of Ibadan, Ibadan, Nigeria; 2 Department of Infectious Disease Epidemiology, London School of Hygiene & Tropical Medicine, London, WC1E 7HT UK; 3 Malaria Consortium, Development House, 56-64 Leonard Street, London, EC24 4LT UK; 4 Department of Health Promotion and Education, College of Medicine, University of Ibadan, Ibadan, Nigeria; 5 Disease Control Department, London School of Hygiene & Tropical Medicine, London, WC1E 7HT UK; 6 Department of Epidemiology and Biostatistics, College of Medicine, University of Ibadan, Ibadan, Nigeria; 7 Department of Pharmacology, College of Medicine, University of Ibadan, Ibadan, Nigeria; 8 Malaria Consortium East and Southern Africa, Plot 2, Sturrock Road, P.O. Box 8045, Kampala, Uganda

**Keywords:** Malaria case management, Malaria capacity building, Malaria intervention, Malaria treatment, Under-five children malaria case management, Malaria presumptive treatment, Support to National Malaria Programme

## Abstract

**Background:**

Despite the uptake of parasitological testing into policy and practice, appropriate prescription of anti-malarials and artemisinin-based combination therapy (ACT) in accordance with test results is variable. This study describes a National Malaria Control Programme-led capacity building intervention which was implemented in 10 States of Nigeria. Using the experience of Niger State, this study assessed the effect on malaria diagnosis and prescription practices among febrile under-fives in rural health facilities.

**Methods:**

The multicomponent capacity building intervention consisted of revised case management manuals; cascade training from national to state level carried out at the local government area (LGA) level; and on the job capacity development through supportive supervision. The evaluation was conducted in 28, principally government-owned, health facilities in two rural LGAs of Niger State, one in which the intervention case management of malaria was implemented and the other acted as a comparison area with no implementation of the intervention. Three outcomes were considered in the context of rapid diagnostic testing (RDT) for malaria which were: the prevalence of RDT testing in febrile children; appropriate treatment of RDT-positive children; and appropriate treatment of RDT-negative children. Outcomes were compared post-intervention between intervention and comparison areas using multivariate logistic regression.

**Results:**

The intervention did not improve appropriate management of under-fives in intervention facilities above that seen for under-fives in comparison facilities. Appropriate treatment with artemisinin-based combinations of RDT-positive and RDT-negative under-fives was equally high in both areas. However, appropriate treatment of RDT-negative children, when defined as receipt of no ACT or any other anti-malarials, was better in comparison areas. In both areas, a small number of RDT-positives were not given ACT, but prescribed an alternative anti-malarial, including artesunate monotherapy. Among RDT-negatives, no under-fives were prescribed artesunate as monotherapy.

**Conclusion:**

In a context of significant stock-outs of both ACT medicines and RDTs, under-fives were not more appropriately managed in intervention than comparison areas. The malaria case management intervention implemented through cascade training reached only approximately half of health workers managing febrile under-fives in this setting. Implementation studies on models of cascade training are needed to define what works in what context.

## Background

In 2010, the World Health Organization (WHO) updated the Guidelines for Malaria Diagnosis and Treatment. The policy of presumptive treatment for malaria in children with no obvious alternative cause of fever was replaced by a recommendation that, where possible, parasitological confirmation of infection be established before treatment, and anti-malarial treatment be restricted to parasite-positive patients [1].

By 2011, 37 African countries including Nigeria had a malaria parasite-based diagnosis for treatment for all age groups [2–5]. Following the introduction of rapid diagnostic tests (RDTs) for malaria, in many African countries there has been a positive trend in the proportion of suspected malaria cases receiving a parasitological test among patients attending the public sector [6]. Among febrile children under 5 years of age surveyed during nationally representative household surveys in the WHO African Region, the prevalence of testing in the public sector was 59% in 2017 [7], and 14% in Nigeria [8].

In 2010, prior to the inception of this study, prevalence of testing was lower still, at about 30% for the WHO African Region as a whole and 5% in Nigeria [7]. However, despite the uptake of parasitological testing into policy and practice, appropriate prescription of anti-malarials and artemisinin-based combination therapy (ACT) in accordance with test results is variable [9]. Studies have shown variability in adherence to current guidelines for malaria treatment based on confirmed diagnosis, in particular in the management of test-negative patients [9, 10]. Parasitological diagnosis is just one of the processes required for a child to receive and benefit from appropriate and effective treatment. The other processes according to the Nigerian National Malaria Treatment guidelines include correct diagnosis, prescription of the most appropriate drug, dispensing of the correct amount of the drugs and clear explanation by the clinician of the diagnosis and dosing regimen. Furthermore, the guidelines emphasize the need for caregiver’s understanding and ability to recall messages relating to adherence to correct dosing regimen [1, 5].

The malaria case management capacity building programme of the Nigerian National Malaria Control Programme (NMCP) took place across a quarter of the States of Nigeria, and this study assessed the effectiveness of the programme on malaria diagnosis and prescription practices among febrile under-fives attending rural health facilities in one of the ten implementing States, Niger State.

## Methods

### Intervention description

Between 2008 and 2015, the Nigerian National Malaria Control Programme was supported to conduct a phased capacity building programme on malaria case management in 10 of the 37 States of Nigeria. These States included: Lagos, Kano, Anambra, Katsina, Niger, Ogun, Jigawa, Enugu, Kaduna and Yobe. Training materials were developed and a system of cascade training from State to local government area (LGA) level (i.e. district equivalent level) was implemented for health care workers.

The malaria case management capacity building intervention consisted of: (1) revised case management manuals including adult learning exercises; (2) cascade training where national level facilitators, including content and adult learning experts, supported the State to train State level facilitators or trainers, who then conducted 3 day training sessions; and (3) on the job capacity development through integrated supportive supervision, with the State level facilitators/trainers acting as supervisors.

### Study area and population

This study was carried out from October 2011 to March 2013, in two local government areas, Katcha and Gbako of Niger State. Niger State is located in the North Central geopolitical zone of Nigeria and has a total of 25 local government areas, with an overall population of approximately 4.3 million people. The State has high infant and under-five mortality rates of 85 and 106 per 1000 live births, respectively, compared with the national average of 75 and 106 per 1000, respectively [3, 11]. Like many other parts of Nigeria, malaria is endemic in Niger State, with year-round transmission. In 2010, 45% of febrile under-fives tested in the North Central zone were RDT-positive [12] and malaria constituted the highest proportion of childhood febrile illnesses presenting to health facilities [3, 11].

At the time of this study, there were 1323 primary health care facilities (PHCs), 18 secondary health facilities and two tertiary health facilities in Niger State. Services, including diagnostics and prescribed treatment were provided free of charge by primary health care facilities to pregnant women and children under-5 years of age. In addition to the government owned health care facilities, there were also 446 registered private health facilities (hospitals, clinics, maternities, and laboratories) and 1200 licensed Patent Medicine Vendors.

### Study design and sampling procedure

The two LGAs were purposively selected on the basis of similarity in socio-economic characteristics and the ratio of public to private sector facilities. Sample size calculation was based on the proportion of under-fives receiving appropriate treatment for febrile illness (i.e. proportion of febrile children treated for malaria or tested for malaria and treated if positive) estimated at 10% in the comparison LGA. Assuming a coefficient of variation k = 0.4, and recruitment of 30 under-fives per facility, a minimum of 28 health facilities (14 per LGA) were required in order to detect an absolute difference in appropriate treatment prevalence between LGAs of 10%, at a power of 80%, and a 5% level of significance (two-sided).

Two stage stratified sampling strategy was used. In the first stage, a full list of all public and private facilities in Katcha and Gbako LGAs was compiled and they were classified as public or private and then into secondary or primary health facilities. Health facilities were selected proportional to the total number of facilities in each LGA. In total 28 facilities were selected, 14 in each LGA. In the second stage 840 febrile under-fives, 30 per facility, were selected from included health facilities. Recruitment of children from facilities continued over subsequent data collection visit days until required sample sizes were achieved.

### Data collection

Surveys were undertaken in Katcha and in Gbako 1 year after implementation of the case management training programme. All health workers involved in management of febrile under-fives on the day of the survey were invited to take part, and following consent, data on demographic characteristics of these staff were collected from the participating health workers and the health facility staff person in charge, using a structured questionnaire. Characteristics of the health facility were collected using a structured questionnaire and health facility stock records were consulted to record availability of supplies and equipment necessary for malaria diagnosis and treatment on the day of the survey. All health facility attendees aged under-5 years presenting with fever or a history of fever on each day of the survey were invited to take part, and following provision of informed consent, participant characteristics for presenting under-fives and their accompanying caregiver were collected and all consultation processes observed and recorded using a structured checklist.

### Statistical methods

Data were collected using paper questionnaires and subsequently double-entered using EpiData version 3.1 (EpiData Association, Odense, Denmark). Data were analysed using STATA version 13.0 (STATA Corporation, College Station, Texas). Sample weights for observations were estimated as the reciprocal of the probability of selection of each health facility for analyses conducted at the health facility level. Additionally, for analyses conducted at the individual under-five patient level, sample weights were estimated based on the product of the sampling weights of each selected health facility and those of individual patients. The probability of selection of individual under-five patients within each study health facility was calculated as 30/mean outpatient attendance for 2010. Descriptive and logistic regression analyses were carried out using svy commands.

Due to the change in national guidelines for malaria diagnosis and the introduction of confirmatory parasitology during the implementation of this intervention, the original primary outcome of appropriate treatment defined as febrile children being presumptively treated for malaria or tested and treated if positive, was no longer considered appropriate. Alternative outcomes of the prevalence of RDT use among febrile under-fives, and appropriate management of RDT-positives and RDT-negatives were generated [13]. Appropriate management was defined as prescription of artemether–lumefantrine (AL) or artesunate–amodiaquine (ASAQ) to RDT-positive under-fives and no AL or ASAQ prescribed to RDT-negative under-fives. As description of the sample showed that many of the RDT-negative under-fives who were not receiving AL or ASAQ were receiving another anti-malarial, appropriate management of RDT-negative was also defined as those RDT-negative under-fives who were not prescribed any anti-malarial.

Comparison of outcomes of clinical malaria diagnosis with rapid diagnostic tests and appropriate management of RDT-positive or RDT–negative under-fives was made between intervention and comparison areas in 2013, following 1 year of implementation of the intervention. Facilities and staff in the intervention LGA received the package of interventions included in the NMCP’s capacity development programme, while the comparison LGA received none of the specified components and experienced only Federal level initiatives (including changes in national policy such as the adoption of the WHO guidelines for the introduction of confirmatory parasitology).

Univariate logistic regression analyses calculated odds ratios (ORs) and confidence intervals (CIs) comparing the odds of these outcomes among under-fives from facilities in intervention and comparison LGAs. In multivariate logistic regression analyses, these associations were investigated controlling for the following a priori defined potential confounders: age and gender of the child; gender and education level of the carer; age and gender of the health worker; as well as RDT availability and either AL or ASAQ availability from health facility stock records [14].

### Ethics

Approval for the study was obtained from the Ethics Committees of the Niger State Ministry of Health, the University of Ibadan/University College Hospital Ethical Review Committee and the Ethics Committee of the London School of Tropical Medicine and Hygiene, UK.

## Results

### Summary of NMCP capacity development programme implementation in Niger State

The implementation of this programme in Niger State began in 2011 at which time surveys with health facility staff caring for febrile under-fives showed that 27% of health workers in intervention area health facilities had reported recent malaria training (in the 6 months before the survey), while 16% had reported recent training in the integrated management of childhood illness (IMCI). Similar levels of training were reported by staff from facilities in the comparison area- 29% for recent malaria training and 23% for recent IMCI training.

By 2013, all 14 facilities in the intervention LGA reported that at least one staff member had attended malaria training, while 13 of the 14 had done so for IMCI training. This contrasted to facilities in the comparison LGA where 12 of 14 facilities reported that at least one staff member had attended malaria training, but 7 of the 14 facilities reported that none of the staff interviewed had recently attended IMCI training. Table 2 shows that a higher proportion (54% versus 34%; p = 0.09) of health workers from health facilities in the intervention LGA reported recent malaria training. In addition, significantly more health workers from facilities in the intervention LGA reported attending IMCI training in the last 6 months (45%), in comparison to 20% from facilities in the comparison LGA. These results suggest that although the training component of the programme reached facilities, cascade training did not fully reach all staff members within intervention facilities. Furthermore, Table 1 shows that in 2013 similar mean numbers of supervision visits were recorded between intervention and comparison LGAs, suggesting that this component of the capacity development programme did not result in more frequent supervision visits in intervention LGAs.

**Table 1:** Health facility characteristics by LGA

	Intervention N = 14			Comparison N = 14			p-value
	N	%	95% CI	n	%	95% CI	
Facility ownership							
Government	12	97.2	86.4, 99.5	13	92.4	54.1, 99.2	
Private	2	2.8	0.5, 13.6	1	7.6	0.8, 45.9	
	**Mean**	**SD**		**Mean**	**SD**		
Number of supervision visits in last 6 months (mean)	2.94	0.51		3.08	1.14		0.91
Catchment area size (mean)	5225	1416		4042	568		0.44

*LGA* local government area, *CI* confidence intervals, *SD* standard deviation

### Description of sample

A total of 28 health facilities were sampled, 14 in each LGA. The large majority were government-owned in both LGAs, with only 3% and 8% of included facilities from the private sector (Table 1). Facilities in both LGAs had similar catchment sizes.

In total, 171 health workers across these 28 health facilities were interviewed and observed during consultations with febrile under-fives during the period of data collection, with 65% of these health workers based in intervention facilities. About 60% of health workers in selected facilities from both LGAs were male, and about 95% from the Nupe ethnic group. Health workers from facilities in both LGAs had a mean age of about 35 years, and about 70% of health workers had worked at that facility for at least 3 years. The majority of staff managing febrile under-fives in this setting were Community Health Officers (CHO) and community health extension workers (CHEWs) (or junior community health extension workers (JCHEWs)), with fewer than 7% of staff managing under-fives in either LGA defined as clinically trained (Table 2).

**Table 2:** Health worker characteristics by LGA

	Intervention N = 111			Comparison N = 60			*p* value
	N	%	95% CI	n	%	95% CI	
Sex (n = 170)							0.784
Male	70	61.1	42.5, 76.9	35	57.3	34.2, 77.7	
Female	40	38.9	23.1, 57.5	25	42.7	22.4, 65.8	
Ethnic group (n = 171)							0.433
Nupe	105	94.6	78.9, 98.8	59	98.2	88.7, 99.7	
Hausa	1	1.0	0.1, 7.2	0	0		
Other	5	4.4	1.1, 15.8	1	1.8	0.3, 11.3	
Highest qualification of health worker (n = 167)							0.191
Physician	2	2.1	0.6, 6.7	0			
Registered nurse	4	4.2	1.6, 10.6	1	1.8	0.3, 11.9	
Registered midwife	0			1	1.8	0.2, 15.0	
Medical assistant	1	1.0	0.1, 7.8	0			
Nursing assistant	0			0			
Laboratory technician	6	5.4	2.1, 13.4	0			
Ward assistant	6	5.4	1.6, 16.4	1	1.8	0.3, 10.9	
CHO	7	5.6	2.0, 14.8	2	3.6	1.0, 11.8	
CHEW	24	21.7	12.9, 34.2	19	32.6	20.6, 47.5	
JCHEW	29	29.6	19.1, 42.7	14	23.7	15.9, 33.7	
Other	32	24.9	14.6, 39.1	21	34.7	23.2, 48.4	
Clinical training (n = 167)							
Clinically trained	6	6.3	2.8, 13.7	2	3.6	0.9, 13.4	0.446
How long worked at facility (n = 171)							0.823
< 1 year	13	10.2	7.0, 14.5	5	7.3	2.4, 20.6	
1–3 years	22	21.4	10.3, 39.1	12	19.7	8.4, 39.6	
> 3 years	76	68.5	52.1, 81.2	43	73.0	55.9, 85.2	
IMCI training (n = 171)	49	44.5	32.4, 57.3	12	19.7	9.0, 37.9	0.020
Malaria training (n = 171)	59	54.1	42.2, 65.5	21	34.1	17.4, 56.0	0.090
	**Mean**	**SD**		**Mean**	**SD**		
Mean health worker age (years) (n = 166)	35.6	1.04		34.8	1.26		0.664

*LGA* local government area, *CI* confidence intervals, *SD* standard deviation, *CHO* Community Health Officers, *CHEW* community health extension workers, *JCHEW* junior community health extension workers, *IMCI* integrated management of childhood illness

In total, 840 febrile under-fives were recruited, 420 from facilities in the intervention LGA and likewise in the comparison LGA. Under-fives in both areas were similar in age, with a mean age of 1.9 years, and similar proportions of boys and girls were recruited. Fewer than 1% of children recruited were covered by the National Health Insurance Scheme. The carers accompanying the under-fives were principally female, the majority the child’s mother. Almost half of carers reported no formal education, and between 22% and 31% reported Islamic education (Table 3).

**Table 3:** Child and carer characteristics by LGA

	Intervention N = 420			Comparison N = 420			p-value
	N	%	95% CI	n	%	95% CI	
Sex of carer (n = 840)							0.379
Male	143	31.7	22.0, 43.4	115	24.9	14.7, 38.9	
Female	277	68.3	56.6, 78.0	305	75.1	61.1, 85.3	
Relationship to child (n = 833)							0.146
Mother	265	63.4	52.1, 73.5	295	73.3	59.3, 83.8	
Father	117	24.2	15.8, 35.2	102	22.5	12.4, 37.4	
Relative	32	10.9	5.9, 19.4	15	3.9	1.9, 7.7	
Other	5	1.4	0.5, 4.1	2	0.3	0.1, 1.6	
Carer education (n = 838)							0.038
None	169	47.1	30.4, 64.4	216	57.9	36.8, 76.4	
Primary	24	4.9	2.3, 10.0	27	6.8	3.8, 12.0	
Middle/junior	10	3.3	1.7, 6.6	3	0.8	0.3, 2.5	
Secondary	70	18.6	11.3, 29.2	11	3.0	1.5, 5.9	
Teritary	27	4.3	2.2, 8.4	1	0.2	0.0, 1.9	
Islamic	120	21.7	10.8, 38.8	160	31.3	14.9, 54.2	
Sex of child (n = 834)							0.990
Male	229	55.2	48.1, 62.0	233	55.2	48.0, 62.1	
Female	189	44.8	38.0, 51.9	183	44.8	37.9, 52.0	
Health insurance (n = 783)	3	0.9	0.1, 5.4	5	1.0	0.2, 5.0	0.956
	**Mean**	**SD**		**Mean**	**SD**		
Mean age child (years) n = 835	1.89	0.15		1.87	0.10		0.994
Mean age carer (years) (n = 464)	29.3	0.42		27.0	1.31		0.107

*LGA* local government area, *CI* confidence intervals’, *SD* standard deviation

### Stock availability

Over the study period, facilities were visited on average 25 times (range 11–32), and stock availability was assessed. On the days of visits, stock availability of RDTs and recommended ACT medicines (AL and ASAQ) were low in facilities from both LGAs. In total, 43% and 15% of included facilities in intervention and comparison LGAs respectively, reported that they had neither AL nor ASAQ available for 10% of the visit days. Stock-out problems for these ACT medicines in the intervention facilities appeared especially binary, with half of facilities reporting no stock on any of the visit days, and the other half reporting availability for over 80% of visit days. On average, over all visit days, AL or ASAQ were only available for about 50% of visit days in both intervention and comparison facilities but, as described above, this was not evenly distributed between all facilities, in particular for those located in the intervention LGA. Problems with RDT stock-outs were also recorded for facilities in both LGAs with 24% and 46% of facilities in intervention and comparison LGAs respectively reporting stock outs of RDTs at every visit day. On average, over all visit days, RDTs were available during 67% of visit days in facilities from the intervention LGA, and during 42% of visits in facilities from the comparison LGA (p = 0.136) (Table 4).

**Table 4:** Stock of essential diagnostics and medicines on the days of the survey by LGA

	Katcha	N = 14	95% CI	Gbako	N = 14	95% CI	p-value
	n	%		n	%		
Proportion of facilities where neither AL nor ASAQ were available on any of the days the facility was visited							
	7	43.3	17.7, 73.1	0			0.031
Proportion of facilities where neither AL nor ASAQ was available for < 10% of the days the facility was visited							
	7	43.3	17.7, 73.1	2	15.2	0.8, 45.9	0.166
Proportion of facilities where RDTs were not available on any of the days the facility was visited							
	3	24.3	6.8, 58.6	6	45.6	19.6, 74.3	0.186
	**Mean**	**SD**		**Mean**	**SD**		
Mean % daily availability of ASAQ	41	14.5		25	8.1		0.337
Mean % daily availability of AL	56	14.3		47	8.9		0.594
Mean % daily availability of ASAQ or AL	56	14.3		50	8.2		0.730
Mean % daily availability of RDTs	67	11.5		42	11.8		0.136

*LGA* local government area, *CI* confidence intervals, *SD* standard deviation, *ASAQ* artesunate amodiaquine, *AL* arthemeter–lumefantrine, *RDT* rapid diagnostic tests

### RDT testing and appropriate treatment for malaria among under-fives

A higher proportion of febrile under-fives were tested with an RDT in facilities from the intervention LGA (56% vs. 36%), but these differences and the effect of the intervention on RDT testing were not statistically significant (adjusted odds ratio (AOR) = 1.41; 95% confidence interval (CI) 0.30, 6.52) (Table 5). Appropriate management of RDT-positive under-fives was high in both areas, with 85% and 90% of RDT-positive under-fives receiving AL or ASAQ in comparison and intervention LGAs, respectively. Multivariate models indicate no difference between the areas (AOR = 1.91; 95% CI 0.36, 10.04) (Table 5).

**Table 5:** Malaria case management outcomes by LGA

Descriptive results								Univariate results				Multivariate results^a^			
	Intervention N = 420			Comparison N = 420			Chi^2^ p-value	N included in model	Crude odds ratio	Lower 95% CI	Upper 95% CI	N included model	Adjusted odds ratio ^a^	Lower 95% CI	Upper 95% CI
	n	%	95% CI	N	%	95% CI									
RDT conducted	253	55.7	28.2, 80.1	153	36.4	16.2, 62.9	0.291	835	2.21	0.48	10.07	823	1.41	0.30, 6.52	0. 650
RDT result	N = 253			N = 153			0.185	NA							
Positive	169	65.3	33.9, 87.3	70	42.2	23.8, 63.1									
Negative	77	34.7	12.7, 66.1	72	57.8	36.9, 76.2									
Appropriate management of RDT positive with ASAQ or AL															
	N = 168			N = 70			0.554								
Yes	153	90.5	35.0, 84.9	63	84.7	60.0, 95.3		238	1.73	0.25	11.93	236	1.91	0.36, 10.04	0.426
Appropriate management of RDT negative (did not receive ASAQ or AL)															
	N = 73			N = 71			0.204								
Yes	66	88.7	66.5, 96.9	67	96.2	82.1, 99.3		144	0.30	0.04	2.21	144	0.68	0.11, 4.36	0.664
Appropriate management of RDT negative (did not receive any antimalarial)															
	N = 73			N = 71			0.004								
Yes	43	37.2	8.9, 78.3	64	92.7	68.0, 98.7		144	0.05	0.005	0.469	144	0.12	0.02, 0.77	0.028

^a^Adjusted for: child characteristics: age, sex; carer characteristics: education, sex; health worker characteristics: age, sex; health facility daily record stock availability characteristics: rdt availability, either AL or ASAQ availability

**Fig. 1:**
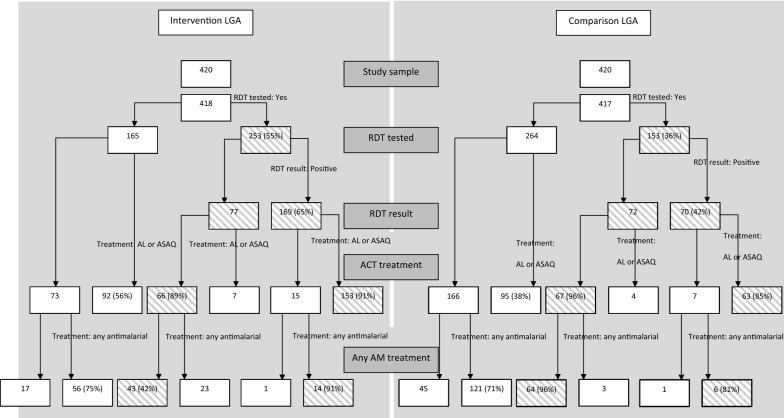
Flow chart of RDT testing, RDT result, Treatment with AL or ASAQ (recommended first line ACT), and Treatment with any anti-malarial

Appropriate management of RDT-negative under-fives was also high in both areas. In total 96% and 89% of RDT-negative under-fives did not receive AL or ASAQ in comparison and intervention LGAs, respectively, and no effect of the intervention on this outcome was detected (AOR = 0.68; 95% CI 0.11, 4.36). However, Fig. 1 shows that, in the intervention LGA, of the 66 RDT-negative under-fives who did not receive the first-line recommended ACTs, 23 (58%) received any anti-malarial, in comparison to 3/67 (4%) in the comparison LGA. When appropriate management of RDT-negative under-fives was defined by those who did not receive any anti-malarial, prevalence of appropriate treatment was 37% in the intervention LGA and 93% in comparison areas. On adjustment for a priori confounders, the odds of appropriate management of RDT-negative under-fives who did not receive any anti-malarial were substantially lower among those children treated at intervention health facilities (AOR 0.12; 95% CI 0.02, 0.77) (Table 5).

In both areas, a small number of RDT-positives (15 in intervention LGA and 7 in comparison LGA) were not given AL or ASAQ, and Fig. 1 shows that these children were given another anti-malarial, frequently artesunate monotherapy or chloroquine. Overall 10% (95% CI 3.8–25.7%) of febrile under-fives in intervention facilities, and 14% (95% CI 5.1–33.5%) in comparison facilities were prescribed artesunate as monotherapy (p = 0.632). Among RDT-positives in both the intervention and comparison facilities, 10% were prescribed artesunate as monotherapy (p = 0.981). However, among RDT-negatives, no under-fives were prescribed artesunate as monotherapy.

## Discussion

The evaluation found there to be a higher prevalence of RDT testing in the health facilities in the intervention LGA (56% *versus* 36%), however this was not statistically significant. This finding is supported by similar results of training involving fever case management from public health centres in Uganda [15], where 68.4% and 41.2% of children were tested in intervention *versus* control facilities adjusted risk ratio 1.66 (confidence interval 0.88, 3.12; p = 0.11). In a synthesis of 10 studies (sites included Afghanistan 2; Cameroon 1; Ghana 1; Nigeria 1; Tanzania 2; and Uganda 3) on the impact of introducing malaria RDTs the ACT Consortium found the proportion of patients tested with an RDT varied widely but that the largest increases in parasitological testing was where RDTs were introduced outside of health facilities [16].

Appropriate management of RDT-positive children aged under 5 years was high with 85% and 90% of RDT-positive under-fives receiving AL or ASAQ in control and intervention LGAs respectively, with no statistical difference between them.

Appropriate management of RDT-negatives was high in both intervention and comparison LGAs when defined as not prescribing an ACT (AL/ASAQ). However, prescription of non-ACT anti-malarials was a problem in intervention LGA health facilities. Based on including non-ACT anti-malarials in the definition of appropriate treatment, the health facilities in the comparison LGA performed better (37% appropriate treatment in intervention versus 93% in comparison health facilities). This finding suggests a particularly high lack of confidence by health workers and/or by patients in negative RDT results in the intervention area. Lack of confidence in and adherence to negative RDT results has been well documented; in a systematic review including seven RCTs, prescribing of anti-malarials to RDT-negative patients was highly variable ranging from 0 to 81% [17]. Qualitative studies have highlighted a range of potential reasons for non-adherence to negative RDT results including those relating to health workers lack of trust in the accuracy of the RDTs; worries on the outcome for the patient with a false negative result; lack of confidence on alternative actions if the fever is not due to malaria; and pressure to be prescribed an anti-malarial from patients [18, 19].

Adherence to appropriate treatment based on RDT results has been reported as relatively high in lower level cadres, such as community health workers [9]. Better adherence to treatment guidelines would, therefore, have been expected than was found. However, similar findings have been reported from Cameroon, where enhanced training effectively reduced inappropriate treatment of RDT-negative children with an anti-malarial from 84 to 31% (RR 0.29, 0.11–0.77; p = 0.02), but basic training had no effect [20].

For an intervention to be successful and expected outcomes to be achieved, the intervention itself needs to have been successfully implemented within a context conducive to its mechanisms leading to its outcomes. Findings on exposure to the malaria case management training intervention suggest that whilst all health facilities were reached, only approximately half of health workers within facilities were. This is an important limitation of cascade training. The intervention did not have an effect resulting in differential health worker behaviour in the intervention and comparison LGAs. There was more training in the intervention LGA, and more in comparison to baseline too but only 50% prevalence of exposure to training amongst health workers. It was not possible to analyse the performance of individual health workers who reported having been trained compared with those not trained, as the question in the survey through which this analysis would be done (health worker id), was poorly completed. The lack of effect does not seem to be likely due to contamination, as IMCI and malaria training decreased in the health facilities in the comparison LGA and the two LGAs were selected as non-bordering.

This study provides an example of the difficulties of implementing an intervention into a routine health system at scale. Cascade training is very commonly used by national programmes faced with the need for training on a large-scale, with limited financial and human resources. There are two major risks in cascade training: firstly of not reaching all of those needing training as in the current study, and secondly, of quality decay as the training is passed down the cascade. Despite a dearth of evidence on the effectiveness of cascade training there are examples of successful models including that of well-supervised cascade-training including refresher workshops to scale-up mental health services in a demonstration project in Nigeria [21]. Training needs to focus on not just initiating activities by health workers but on embedding these activities in their working day [22]. More implementation studies are needed on different models of cascade training in order to ensure their effectiveness and maximize their potential.

## Limitations

The originally planned pre-post comparison study design was not possible due to policy change, requiring a change in definition of the outcome of appropriate treatment to include RDT testing, which was not available at baseline. The baseline survey was undertaken pre-policy change from presumptive treatment of febrile children with an ACT to parasitological diagnosis, with treatment based on this diagnosis. No record of RDT use in either LGA was taken during the baseline survey.

## Conclusions

The study did not show a significant difference in the use of parasitological testing for malaria in febrile children in intervention and comparison health facilities. The malaria case management intervention implemented through cascade training reached approximately half of health workers in a context of significant stock-outs of both ACT medicines and RDTs.

## Data Availability

Data for this study are available with the authors at both the London School of Hygiene and Tropical Medicine and the University of Ibadan. They are not publicly available for confidentiality.

## Abbreviations

ACT: Artemisinin-based combination therapy
ASAQ: Artesunate–amodiaquine
AL: Artemether–lumefantrine
CIs: Confidence intervals
CHEWs: Community health extension workers
CHO: Community Health Officers
IMCI: Integrated management of childhood illness
JCHEWs: Junior community health extension workers
LGA: Local government area
NHIS: National Health Insurance Scheme
NMCP: National Malaria Control Programme
ORs: Odds ratios
PHCs: Primary health care facilities
RDTs: Rapid diagnostic tests
SD: Standard deviation
WHO: World Health Organization
